# Bone Effects of Anti-Cancer Treatments in 2024

**DOI:** 10.1007/s00223-025-01362-0

**Published:** 2025-03-27

**Authors:** Marie Teissonnière, Mathieu Point, Emmanuel Biver, Peyman Hadji, Edith Bonnelye, Peter R. Ebeling, David Kendler, Tobias de Villiers, Gerold Holzer, Jean-Jacques Body, Ghada El Hajj Fuleihan, Maria Luisa Brandi, René Rizzoli, Cyrille B. Confavreux

**Affiliations:** 1 Pharmacie, Hôpital Lyon Sud, Hospices Civils de Lyon, Pierre-Bénite, France; 2 INSERM UMR1033–LYOS, Université Claude Bernard Lyon 1, Université de Lyon, Lyon, France; 3 Division of Bone Diseases, Geneva University Hospitals and Faculty of Medicine, University of Geneva, Geneva, Switzerland; 4 Frankfurt Center of Bone Health & Philipps University of Marburg, Frankfurt, Germany; 5 Univ. Lille, CNRS, Inserm, CHU Lille, UMR9020-U1277-CANTHER–Cancer Heterogeneity Plasticity and Resistance to Therapies, Lille, France; 6 Department of Medicine, School of Clinical Sciences, Monash University, Clayton, VIC 3168 Australia; 7 Department of Medicine (Endocrinology), University of British Columbia, Vancouver, Canada; 8 Department Gynaecology, Stellenbosch University, Cape Town, South Africa; 9 Medical University of Vienna, Vienna, Austria; 10 Department of Medicine, CHU Brugmann, Université Libre de Bruxelles, Brussels, Belgium; 11 Calcium Metabolism and Osteoporosis Program WHO Center for Metabolic Bone Disorders, American University of Beirut, Beirut, Lebanon; 12 FIRMO Foundation, Florence and University Vita-Salute San Raffaele, Milan, Italy; 13 Geneva University Hospitals and Faculty of Medicine, Geneva, Switzerland; 14 Rheumatology Department, Bone Metastasis Expert Center (CEMOS), Hospices Civils of Lyon Cancer Institute (IC-HCL), Hôpital Lyon Sud, Pierre-Bénite, France; 15 Centre Expert Des Métastases Osseuses (CEMOS), Service de Rhumatologie Sud, Hôpital Lyon Sud, 165 chemin du Grand Revoyet, 69310 Pierre Bénite, France

**Keywords:** Cancer treatment induced bone loss (CTIBL), Bone mineral densitometry, Side effects, Fracture risk, Osteoblast, Osteoclast

## Abstract

Considerable progress has been made in the management of cancer patients in the last decade with the arrival of anti-cancer immunotherapies (immune checkpoint inhibitors) and targeted therapies. As a result, a broad spectrum of cancers, not just hormone-sensitive ones, have seen several patients achieve profound and prolonged remissions, or even cures. The management of medium- and long-term side-effects of treatment and quality of life of patients are essential considerations. This is especially true for bone, as bone fragility can lead to increased fractures and loss of autonomy, ultimately reducing the possibility of resuming physical activity. Physical activity is essential for lasting oncological remission and prevention of fatigue. While the issue of hormone therapies and their association with breast cancer has been recognized for some time, the situation is relatively new with regards to targeted therapies and immunotherapies. This is particularly challenging given the wide range of available targeted therapies and their application to numerous cancer types. This article provides a comprehensive review of the bone effects of the main anti-cancer therapies currently in use. The review goes beyond glucocorticoids and hormone therapies and discusses for each drug category what is known regarding cellular effects, BMD effects, and fracture incidence.

**Supplementary Information:**

The online version contains supplementary material available at 10.1007/s00223-025-01362-0.

## Introduction

In the last decade, oncology and hematology fields underwent drastic improvements in cancer care with the onset of novel non-chemotherapy drugs. This has contributed to the transformation of cancer into a chronic disease in an increasing number of patients across all tumor types even in metastatic stages. Beyond survival itself, issues of long-term quality of life in cancer patients becomes increasingly important. In particular, the issue of long-term bone health was a quite common parameter in breast and prostate cancers patients on hormonal therapy. Nowadays this question may be expanded to many other cancer types including pediatric cancers [1–3].

Mobility is one of the major items involved in autonomy and quality of life in patients. This mobility relies on bone strength, muscle strength, healthy joints and movement coordination.

Bone strength comprises two components, bone mass and bone quality that may be impaired during cancer treatment. The paradigm of cancer treatment-induced bone loss (CTIBL) is the one observed during hormonal deprivation. Most upcoming drugs in oncology and hematology are not chemotherapy drugs but signaling pathway modulators in cancer cells. Most of bone effects of these long-term treatments remain unknown and to our knowledge, there is no recent review dealing with bone effects of these new drugs used in cancer management. Thus, there is a need to review in adults, the beneficial and adverse bone effects of newer drugs used in cancer management. In childhood cancer patients, the drugs used are associated with particular adverse effects due to their effects on growth and bone development of children, with subsequent consequences as they grow into adults [1, 2]. This manuscript will not consider bone fragility encountered in metastatic bone disease nor associated measures for bone health prevention [3].

## Materials and Methods

**Fig. 1:**
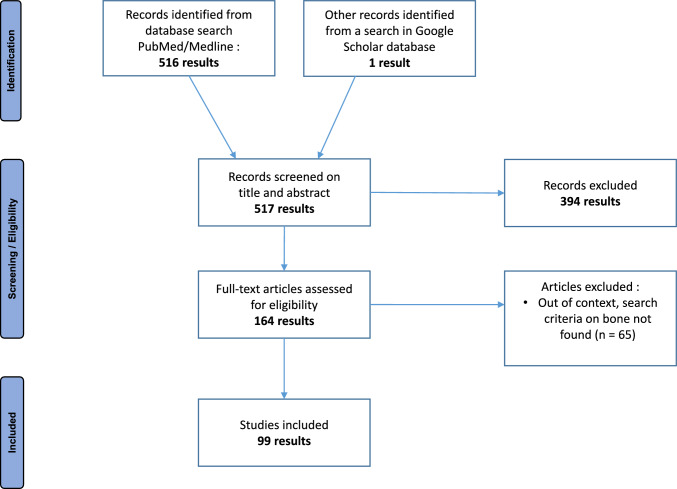
PRISMA flow diagram for identification, screening/eligibility, and inclusion of the studies

We performed a systematic literature research. The search criteria were in vitro*, *in vivo and human studies of the effects of anti-cancer drugs on bone mineral density (BMD), fracture risk and bone cells (osteoblasts and osteoclasts) mainly in adults. Drugs included in the research were corticosteroids, hormone therapy drugs, proteasome inhibitors, tyrosine kinase inhibitors, anti-VEGF antibodies, immune checkpoint inhibitors, mTor inhibitors, anti-angiogenics and cytostatics. The keywords used on Pubmed/Medline were: ("Bone and Bones/drug effects" [Mesh] OR "Bone and Bones/pathology" [Mesh] OR "Bone Remodeling/drug effects" [Mesh] OR "Osteoblasts" [Mesh]) AND ("Antineoplastic Agents/adverse effects" [Mesh] OR "Antineoplastic Agents/drug effects" [Mesh] OR "Antineoplastic Agents/drug therapy" [Mesh] OR "Antineoplastic Agent" [TiAb] OR "Anticancer Agent" [OR "Chemotherapy Agent" [TiAb] OR "Antitumor Agent" [TiAb] OR "Protein Kinase Inhibitors" [Mesh] OR "Protein Kinase Inhibitors" [TiAb] OR "Proteasome Inhibitors" [Mesh] OR "Proteasome Inhibitor*" [TiAb]). One article, not referenced on Pubmed/Medline but in Google Scholar, has also been selected. The last literature search has been performed on August 2023. Anti-resorptive agents and odanacatib have been excluded from the search since they have direct bone effects but they are not considered as anti-cancer drugs themselves. The bibliographic search identified cases, studies, reviews and meta-analyses without limitation. All non-English references were excluded. Figure 1.

## Results

### Glucocorticoid Therapy

In oncology, glucocorticoids (dexamethasone, prednisone, prednisolone) are used in high doses and as background, support or adjuvant treatment to chemotherapy either to potentiate the effects of anticancer drugs or to prevent adverse effects. Glucocorticoids are also known to induce effects on bone cells. Most BMD and fracture risk data have been obtained in non-cancer inflammatory chronic diseases. The use of glucocorticoids is different between oncology and inflammatory chronic diseases. Inflammatory chronic diseases require usually long term use with the search of the minimum effective dose and slow reduction in stages. By contrast, in oncology and hematology, glucocorticoids are prescribed on a high dose intermittent basis with various objectives during chemotherapy: anti-nausea, anti-allergic, anti-oedematous and anti-tumoral. Bone effects are more severe when glucocorticoid is prolonged and the daily dose is high, leading to a high cumulative dose. Thus, a dose of more than 7.5 mg/day – and for some even 5 mg/day- of oral prednisone or equivalent daily, exceeding 3 months of treatment is the commonly agreed upon threshold for considering glucocorticoids as risk factor for osteoporosis [4]. Nevertheless, it is a continuum and there may be individual variations but there is no trivial glucocorticoid dose for bone. Regular intermittent high dose regimens in oncology have been less studied. The worst case-scenario would probably be the long term weekly regimen of high dose dexamethasone in elderly with multiple myeloma that already have a severe bone fragilization. Another frequent side-effect is glucocorticoid-induced epiphyseal bone osteonecrosis [5]. Moreover, in cancer setting, glucocorticoids have also been involved as an additional risk factors of osteonecrosis of the jaw in bone metastatic patients treated with high dose intensity of antiresorptive agents. Altogether, there is interaction between underlying bone health, co-treatments and the glucocorticoids. Bone toxicity of glucocorticoids has been largely reviewed elsewhere [4, 6]. Key points are summarized below.

#### Risk of Fracture

Fracture risk increases early, as soon as 3–6 months after initiation [7]. Notably, the vertebral fracture risk is particularly increased [8]. Although fracture risk is related to the glucocorticoids dose, lower doses also have an effect (+ 20% at 5 mg/day compared to + 60% at 20 mg/day) [7, 8].

For a similar BMD value, the relative risk of any fracture in patient on glucocorticoids ranged from 1.98 at the age of 50 years to 1.66 at the age of 85 years in comparison with untreated patients. For hip fracture, the risk is 4.42 and 2.48, respectively [9].

Fracture risk may be high, even if BMD loss is not severe. Physicians should be aware of this discrepancy and consider the cumulative doses, duration of exposure and the persisting drug exposure [1]. Cumulative doses are correlated with an increased risk of vertebral and non-vertebral fractures, particularly at the hip and forearm [7]. These effects appear to be reversible within one year of cessation of treatment, regardless of underlying pathology, age, or gender [4].

#### Effect on BMD

In adults, glucocorticoid therapy is associated with increased bone loss from the first few months and in a dose-dependent manner. Return to normal values upon glucocorticoid withdrawal is not universal, and usually, there is only a partial recovery. Nevertheless, with glucocorticoid therapy there is a discrepancy between the magnitude of BMD changes and the severity of bone strength impairment.

Adolescents may be subject to very high doses, ≥ 900 mg/m^2^ prednisone equivalent. Compared with adults, adolescents are more likely not to achieve peak bone mass and to develop bone loss due to the effect of glucocorticoids on bone formation and bone growth [10]. Nevertheless, some data from nephrology studies with such high doses are reassuring in showing that despite their shorter stature, children have no deficit in bone mineral content of the spine or whole body after adjustment for age, bone size, sex, and degree of maturation [11].

Of note, different types of glucocorticoids differ in potency and side effects. In particular dexamethasone, one of the most powerful glucocorticoid regularly used in multiple myeloma, is associated with a much higher risk of bone loss compared with other glucocorticoids throughout the duration of treatment [10, 12].

#### Effect on Bone Cells and Metabolism

Glucocorticoid has negative effects on osteoblast physiology. Indeed, on osteoblasts, the bone forming cells, dexamethasone inactivates the transcription factors Osterix and Runx2, involved in osteoblastic differentiation. At high doses, dexamethasone decreases regulatory pathways involved in proliferation and differentiation such as Wnt/β-catenin, BMP and Notch [10]. An indirect pathway has also been described through the stimulation of sclerostin that is secreted by osteocytes and which is one of the main physiological inhibitors of Wnt/β-catenin osteoblast anabolic pathway. Indeed, dexamethasone acts on sclerostin promotor that has several glucocorticoid response elements [13]. Thus, dexamethasone administration enhances sclerostin expression that inhibits Wnt pathway and mineralization. Interestingly, *sclerostin* deletion was associated with a marked loss of dexamethasone inhibition effect on bone formation suggesting that sclerostin was a key player in steroid-induced osteoporosis [13]. Furthermore, dexamethasone activates the TAK1 signaling pathway, which is involved in apoptotic events in both osteocytes and osteoblastic cells [14].

In addition, high doses of glucocorticoids increase the number and activity of osteoclasts, the bone resorbing cells, by increasing RANKL synthesis and suppressing osteoprotegerin (OPG) levels shifting the ratio in favor of osteoclastogenesis [10].

Glucocorticoid therapy impacts markers of bone turnover by markedly reducing bone formation markers (bone alkaline phosphatase (ALP), osteocalcin, P1NP) and enhancing bone resorption markers such as serum CTX. For example, dexamethasone suppresses ALP during the intensification period of treatment of acute lymphocytic leukemia. Next, ALP partially normalizes after discontinuation. In addition, the use of high-dose glucocorticoids during the induction and intensification phases is associated with an altered longitudinal bone growth. This growth impairment is significantly more severe with dexamethasone [10].

### Hormone Therapy

Estradiol and, to a lesser extend, testosterone, are sex steroid hormones essential for bone development, growth and maintenance. These hormones naturally synthesized by the body, have numerous effects on various tissues. Some tumors expressing steroid hormone receptors are called hormone-sensitive since their growth is related to these hormones. In breast cancers, estrogen is a key factor for cancer cells. Estrogen is not only produced by ovaries but also in adipose tissue by peripheral conversion of androgens into estrogens (aromatization). This peripheral conversion persists after menopause. In addition, breast cancer cells are also able to synthesize estradiol, independent of circulating serum estradiol levels [15].

In men, testosterone and dihydrotestosterone are two major androgens, essential for normal prostate development and maintenance of physiological functions. Testicles, ovaries and adrenal glands are the organs that produce most of these androgens. Just like breast cancer, prostate tumor cells can synthesize the enzymes involved in androgen synthesis, allowing them to survive, grow and resist to hormones deprivation therapies.

Therefore, in hormonal sensitive breast and prostate cancers, the treatment goal is to fully block this synthesis by either surgical means, radiotherapy or pharmacological castration. Pharmacological drugs act either through a central or peripheral blockade. In central blockade, the hypothalamo-hypophyseal GnRH/LH-FSH axis is blocked. This can be achieved by GnRH antagonists or continuous GnRH agonists to induce a decrease in the production of pituitary FSH/LH resulting in a decrease in circulating estradiol/testosterone concentrations. Onset of action is rapid, within the first week (Table 1). GnRH agonists are used in premenopausal breast cancer and allow ovarian conservation, although the use of GnRH agonists sequentially or concurrently with chemotherapy shows no significant difference in all-cause survival [16, 17].

**Table 1:**
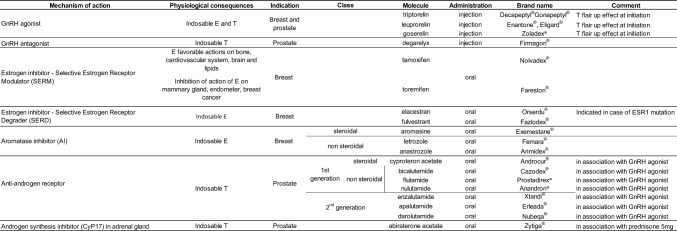
Treatments used as hormonotherapy in cancer

In men with prostate cancer, all-cause survival is improved by GnRH agonist therapy in locally advanced forms of prostate cancer [18]. In a meta-analysis, compared with agonists, GnRH antagonists are associated with improved all-cause mortality (RR: 0.48, 95% CI: 0.26–0.90, *p* = 0.02) but there is no difference on prostate-specific antigen (PSA) progression over a short follow-up period [19]. Peripheral blockade may be achieved by antagonizing androgen/estrogen receptors; or by blocking peripheral conversion (aromatase inhibitors).

#### GnRH Analogs

##### Risk of Fracture

Between 1 and 5 years after cancer diagnosis, the risk of fracture is 19.4% under GnRH agonist treatment and 12.6% without treatment and increases with length of treatment. This excess risk of fracture (RR = 1.21) is associated with increased mortality [20, 21]. In 218 men on GnRH agonists, the mean age of onset of fractures was 78 years with a mean time to onset of 28 months and consistently associated with a decrease in BMD [22]. The rate of musculoskeletal events including fracture risk is significantly higher with GnRH antagonists. One treatment strategy is to offer a GnRH antagonist for a short period of time followed by a GnRH agonist to avoid a large increase in testosterone and the use of anti-androgens [19].

##### Effect on BMD

**Fig. 2:**
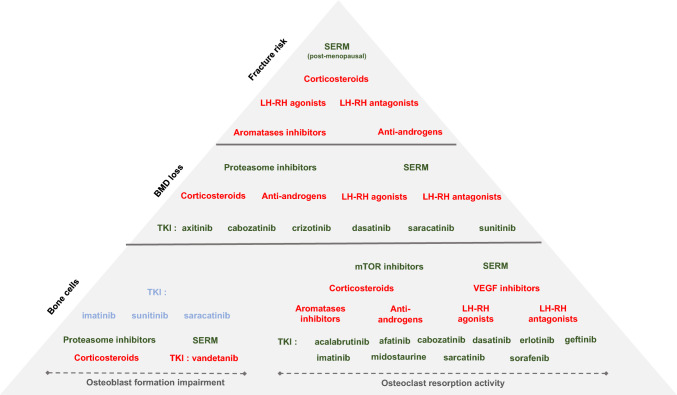
Schematic representation of bone effects of anti-cancer drugs according to three levels of evidence: osteoblast and osteoclast, bone mineral density and fracture. BMD: Bone Mineral Density, SERM: Selective Estrogen Receptor Modulator, TKI:Tyrosine Kinase Inhibitor 5-FU: 5-fluorouracil and AI: aromatase inhibitor. In red: increase, in green: decrease and in blue: neutral

Available data on BMD are more extensive in women with breast cancer than in men with prostate cancer. At low or high doses, GnRH antagonists are associated with a decrease in BMD as early as week 12 of treatment [23]. Figure 2.

In premenopausal women on GnRH analogues monotherapy, a decrease in BMD of 8.2%/year of the lumbar spine is observed and 9.3%/year when combined with aromatase inhibitors (AIs) [24]. In men, bone loss is significant, especially during the first year of treatment. This trend is observed as long as the subject is on treatment but is even more severe in the first year of treatment when castration is complete and sudden. Upon discontinuation, an improvement in bone loss is observed but this is usually only partial [20, 25, 26]. The therapeutic strategy of intermittent administration reduces the risk of progressive bone loss [27]. In children, GnRH agonists induce bone loss in the trabecular and cortical bone as early as 6 months of age. Upon discontinuation, reversibility is only partial [1].

##### Effect on Bone Remodeling

The hormonal deficit caused by GnRH analogues has an impact on bone remodeling [23]. This is more pronounced with GnRH agonists than with anti-androgens and involves an acceleration of bone remodeling as shown by urinary NTX or osteocalcin [27, 28]. With GnRH antagonists, a dose-dependent increase in serum CTX is observed within the first 6 months. GnRH agonists and antagonists also increase bone resorption and accelerate loss of bone strength [23].

#### Anti-Androgens

Blocking the hypothalamo-hypophyseal axis alone is generally not sufficient to completely block androgen production and induce complete castration in men. This is why a combination of anti-androgens is frequently used. There are steroidal anti-androgens (cyproterone acetate and abiraterone) and non-steroidal anti-androgens (enzalutamide, bicalutamide, apalutamide, flutamide, nilutamide and darolutamide). Abiraterone is a selective inhibitor of P450 C17, a key enzyme in the synthesis of testosterone, allowing the blockage of its production. It is indicated in hormone-sensitive and castration-resistant metastatic forms. Cyproterone is a competitive inhibitor of 5-dihydrotestosterone binding to its receptor. The other non-steroidal anti-androgens are androgen receptor inhibitors. Compared with bicalutamide, the other molecules have a higher affinity for their target without exerting an agonist effect. They are indicated in prostate cancer metastatic or non-metastatic forms, hormone-sensitive or not, depending on the molecule.

##### Risk of Fracture

Incidence of fractures in patients on anti-androgens is 7.3% at 24 months (vs. 1.1% in control group) corresponding to a sevenfold higher incidence. The difference was already significant after 12 months of follow-up [18]. At 5 years, epidemiologic and retrospective studies indicate that approximately 20% of men receiving anti-androgens will have an osteoporotic fracture [29]. With new generation (or non-steroidal) anti-androgens, the occurrence of fractures is 6.3% (vs. 4.6% with placebo) with apalutamide [30], 6.5% (vs 4.2% with placebo) with enzalutamide [31] and 4.2% (vs. 3.6% with placebo, ns) with darolutamide [32, 33]. Fracture risk has been computed in a meta-analysis of 519,168 men (16 studies) by Wu et al. They report that androgen deprivation use is associated with an increased fracture risk of nearly 40% (OR, 1.39; 95% CI, 1.26–1.52). OR for fracture requiring hospitalization is also increased (OR, 1.55; 95% CI, 1.29–1.88) [34].

##### Effect on BMD

The extent of bone loss depends on the duration of treatment and whether the anti-androgens are administered continuously or discontinuously. Decrease in BMD is rapid, usually observed as early as 9 to 12 months of treatment [35, 36]. For example, at 1 year, a decrease in BMD on the spine between 2% and 8% and on the femoral neck between 1.8% and 6.5% is observed. Beyond 2 years of treatment, BMD continues to decrease under treatment [29]. The incidence of osteoporosis (T-score ≤ -2.5) is highest in the distal forearm and femoral neck [37]. Recovery is dependent on testosterone levels at cessation of treatment. Other studies, on the other hand, have shown the protective role of bicalutamide on BMD [38]. Compared with untreated men, postmenopausal women and women treated with aromatase inhibitors, bone loss is greater in men treated with anti-androgens.

##### Effect on Bone Cells and Remodeling

Urinary markers of bone turnover are significantly increased by anti-androgens [37]. In vitro, inhibition of osteoclastogenesis with down-regulation of osteoclastic markers (TRAP, κ-cathepsin, metalloproteinase-9) and up-regulation of osteoblastic markers (ALP, osteocalcin) are observed. In vitro and in vivo, enzalutamide decreases the expression of TGFBR2 on the osteoblast, which is responsible for the development of bone lesions [39]. The effects on bone of apalutamide, darolutamide, flutamide and nilutamide have not yet been studied.

#### Anti-Estrogens

Anti-estrogens bind to estrogen receptors, blocking the binding of natural estrogen to its receptor. There are 2 types of anti-estrogens: "Selective Estrogen Receptor Modulators" (SERM) that are competitive inhibitors of estradiol binding to the receptor (tamoxifen, toremifene and raloxifene) and "Selective Estrogen Receptor Degraders" (SERD) that bind to the ER and cause the ER to be degraded and thus downregulated (fulvestrant).

##### Risk of Fracture

Over a long-term period, a 32% reduction in the risk of fracture over 7 years was observed with tamoxifen (RR = 0.68; 95%CI 0.51–0.92) and 50% over 2 years with toremifene. This effect is observed at low doses at 20 mg/day [40–42]. Interestingly, a differential effect seems to exist between premenopausal and postmenopausal breast cancer women. In a case control study (*n* = 5520 per group), there is an increased fracture risk in tamoxifen treated premenopausal women vs. untreated women (6.3% vs 3.6%; *p* < 0.001). This is not observed in the postmenopausal women (10.1% vs 9.3%; ns) [43]. Indeed this is due to a differential effect of SERM according to estrogen impregnation. In low estrogen impregnation (post-menopausal), SERM have an agonist effect on bone while in regular estrogen impregnation (pre-menopausal), SERM have a competitive action with estrogen that decreases bone mass [43]. Fracture risk on fulvestrant has not been studied.

##### Effect on BMD

Tamoxifen and toremifene preserve or even increase BMD of the lumbar spine, hip, and femoral neck at 2 years in postmenopausal women with breast cancer [42]. In 140 postmenopausal women, tamoxifen increased BMD by 0.6% vs. placebo group (1% loss) [44]. In premenopausal women, a decrease in BMD of 1.4% was observed at 3 years vs. a gain of 0.2% in the placebo group. This was also reported by Powles et *al.* with a mean annual loss over 3 years of 1.4%/year at lumbar spine vs. a gain of 0.3%/year in placebo in premenopausal women whereas a significant gain was observed in postmenopausal women (1.2%/year) on tamoxifen [45]. As for fracture risk, the discrepancy between pre- and post-menopausal women is explained by the SERM mechanism of action in presence (premenopausal) or in absence (postmenopausal) of estradiol. In absence of estradiol, SERM acts as an agonist on bone and thus prevents bone loss. By contrast in premenopausal situation, since SERM is less potent than estradiol, it acts as a partial antagonist competitor of estradiol leading to bone loss [24, 46]. In combination with gosereline, the decrease at 2 years in total BMD is lower compared with goseroline alone (-5% goseroline alone vs. -1.4% goseroline + tamoxifen) [47]. The effect of BMD on fulvestrant has not been studied.

##### Effect on Bone Remodeling

Alone or in combination with AIs, tamoxifen decreases bone turnover [42, 48]. Urinary NTX decreases rapidly in the first few months and then gradually over the course of treatment. Bone turnover markers are significantly lower at 1 and 2 years of treatment compared to AIs. Bone ALPs are significantly lower at 2 years of treatment vs. on AI [49].

#### Aromatase Inhibitors

Aromatase is an enzyme involved in the conversion of androgens to estrogens in postmenopausal women. Aromatase inhibitors block the activity of this enzyme, preventing the binding of estrogens to the receptors of tumor cells and consequently their growth. The most commonly used are exemestane (steroidal), anastrozole and letrozole (non-steroidal).

##### Risk of Fracture

Third generation AIs are associated with an increased risk of osteoporosis and fractures in breast cancer studies in comparison with SERMs or placebo [50–55]. For example, in the BIG 1–98, fracture incidence on letrozole was significantly higher on AI than tamoxifen (8.6% vs 5.8%, respectively; *p* = < 0.001) [56]. This was the same with the ABCSG study [54, 57]. The difference is significant even if the protocol starts with tamoxifene for 2 or 3 years before introducing the exemestane [58]. In RCT, the gap with AI seems to be higher with tamoxifen, that has a small protective bone effect, than with placebo [59]. In real life, based on Swedish National registers, Colzani et al*.* also highlighted a trend of excess risk of fractures under AI in comparison with tamoxifen (HR = 1.48; 95%CI 0.98–2.22) [60].

Interestingly, long term follow-up at 100 months of the ATAC study confirms this higher fracture rate with anastrozole compared with tamoxifen (2.93% vs. 1.9%; p < 0.0001) during the time on therapy [61]. Nevertheless, upon treatment discontinuation, the increased fracture risk is not persistent over the long term suggesting bone loss remains partially reversible [61–63].

A meta-analysis has been performed by Goldvaser et al*.* including 16,349 postmenopausal women from 7 trials [64]. The odds ratio for bone fractures is OR = 1.34; 95%CI 1.16–1.55. There is no association between relative OR and median age at random assignment, median duration of follow-up, or the proportion of women previously treated with tamoxifen or chemotherapy. Absolute fracture risk is increased on AI (6.3%) with an absolute difference of + 1.39%.

##### Effect on BMD

Bone loss is evidenced in the spine and hip higher on AI compared with SERMs [42, 52, 65, 66]. AI bone loss is related to the collapsed residual estradiol levels [67]. Therefore, these effects are also most pronounced in the 10 years after the onset of menopause [26, 42, 63, 67, 68]. All AIs cause a comparable, rather constant decrease of BMD at all sites; likewise, fracture risk is equivalently increased as shown in MA 27 study [69]. Direct comparisons of BMD loss between AIs are scarce but there is no significant difference between anastrozole and exemestane on BMD at 2 years [49, 63]. Nevertheless, in animals, some studies report a difference between AI class with non-steroidal AIs inducing less bone loss in comparison with steroidal AIs due to the mechanism of action [70]. Interestingly, in the long-term bone loss analysis of anastrozole vs. tamoxifen, a partial regression was observed upon treatment discontinuation [26].

In addition to aBMD, the MAP.3 study has performed high-resolution peripheral quantitative CT at the distal radius and tibia in non-osteoporotic post-menopausal women receiving exemestane vs. placebo. After 2 years on exemestane, total volumetric BMD declines (-6.1% vs. -1.8% and -5.0% vs. -1.3% at the radius and the tibia, respectively). Cortical thickness is also altered (-7.9% vs -1.1% and -7.6% vs -0.7% at the radius and the tibia, respectively) [71].

##### Effects on Bone Remodeling

AIs are associated with elevated markers of bone resorption and increased bone turnover [42, 49]. These markers (urinary NTX, bone ALP) increase early in treatment and then levels stabilize [49]. Upon discontinuation and concomitant with improved BMD, bone remodeling decreases, mineralization and matrix volume increase [26].

#### Preventing Hormonal Therapy Induced Bone Loss

Several studies have demonstrated the efficacy of oral bisphosphonates to prevent hormonal therapy induced bone loss particularly with aromatase inhibitors in breast cancer as summarized by Rizzoli et al. in a systematic review [72] and the meta-analysis of Su [73]. Zoledronic acid (ZA), an intravenous bisphosphonate, has also demonstrated its efficacy versus placebo to prevent bone loss in premenopausal women with studies like ProBONE II (ZA 4 mg/3 months regimen) and ABCSG 12 (ZA 4 mg/6 months regimen) [74, 75]. In postmenopausal women, strategy studies testing immediate versus delayed ZA also demonstrated the impact of an early ZA treatment to prevent induced bone loss, particularly in recently post-menopausal women (ZO-FAST, Z-FAST) [76, 77]. Denosumab (60 mg/6 months) has been also evaluated and demonstrated a significant gain of BMD in post-menopausal women treated by aromatase inhibitors [78–80]. Moreover, a significant fracture risk reduction has been observed in ABCSG 18 [80]. In addition, a significant antitumor benefit (recurrence, distant recurrence, bone recurrence and breast cancer mortality) has been demonstrated with adjuvant bisphosphonates in postmenopausal women with breast cancer [81]. Prevention of castration induced bone loss in men suffering from hormone sensitive prostate cancer has also been demonstrated for ZA [82–85] and oral bisphosphonates [86–88]. Altogether these results contributed to the current ESMO guidelines to prevent hormonal therapy induced bone loss [89].

### Proteasome Inhibitors (PIs)

Ubiquitin–proteasome system has an essential role in controlling cell division, signal transduction, apoptosis and protein quality enhancement. The targets of proteasome-dependent proteolysis may be various oncogenes/antioncogenes such as p21 and p27 proteins, tumor suppressor proteins p53, RB (retinoblastoma protein), BCL-2, MYC, FOS and JUN family of oncogenes, E2A, E2F, STAT transcription factors and NF-kB transcription factor inhibitors. The ultimate goal of PIs (carfilzomib, bortezomib and ixazomib) is to induce tumor cell death and improve survival in myeloma [90], mantle cell lymphoma [91]. In terms of efficacy, PIs are comparable [92].

#### Risk of Fracture

Fracture risk of PIs has not been studied in humans. In mice, bortezomib promotes fracture healing in young mice and the combination with bisphosphonates promotes fracture healing in old mice [93].

#### Effect on BMD

A significant increase in BMD is detected at axial sites under bortezomib and with an increase in trabecular bone volume under ixazomib [94, 95].

#### Effect on Bone Cells

On the osteoblast, PIs stimulate osteoblastic differentiation without impacting the number of osteoblastic progenitors and the viability of mature osteoblasts [94–96]. This activation is enabled by positive modulation of proteolytic degradation of Runx2/Cbfa1 transcription factors, through inhibition of *Dkk-1* gene expression but without impacting the canonical signaling pathway. Bortezomib also significantly increases the expression of *DLX-5*, a transcription factor involved in osteoblast development [94]. Finally, ixazomib stimulates osteoblastogenesis from mesenchymal stem cells as it increases the expression level of BMP-2 mRNA [96].

PIs promote bone formation through activation of β-catenin signaling pathways and induction of TCF transcriptional activity on osteoblastic and stromal cells [94]. The level of bone formation markers correlates with treatment response [95]. In vitro, bortezomib induces a moderate though not significant increase in ALP after 4, 7 and 14 days [94].

On osteoclasts, PIs are potent osteoclastogenesis inhibitors by intervening at the early and late stages of osteoclast differentiation without affecting precursor viability. This inhibition is dose-dependent and enabled by modulation of the signaling pathway involving p38, RANK-induced NF-κB signaling pathways, and transcriptional factor AP-1 [94–96]. MIP-1α is a key component of osteoclastogenesis, expressed by osteoclasts and myeloma cells. Their concentration correlates with the presence of osteolytic lesions. Bortezomib also inhibits MIP-1α, a key component of osteoclastogenesis [94].

### Tyrosine Kinase Inhibitors (TKIs)

Membrane receptors with tyrosine kinase activity and cytoplasmic tyrosine kinases are important therapeutic targets in cancer treatment, due to their involvement in cell proliferation and survival [97–99]. TKI can inhibit one or several tyrosine kinases leading to different inhibition profiles and have become essential tools for eligible tumors and allow to introduce personalize medicine for patients (Supplemental Table 1).

#### Effect on BMD and Fracture

Available data on the bone effect of TKIs on BMD and bone cells are limited and summarized in Table 2. There are no data in humans either on BMD or on fragility fracture risk. In mice, data are very scarce. A group of reported TKIs increase BMD: axitinib, cabozantinib, crizotinib, dasatinib, saracatinib, nilotinib. This was not the case for sunitinib, which decreases BMD [100, 101].

**Table 2:** Available data on bone tyrosine kinase inhibitory effects on bone mineral density and bone cells

Drugs	BMD	Bone cells
Abemaciclib	uk	uk
Acalabrutinib	uk	OC: inh
Afatinib	uk	OC: inh
Alectinib	uk	uk
Alpelisib	uk	uk
Axitinib	+ (mice)	uk
Bosutinib	uk	0
Brigatinib	uk	uk
Cabozantinib	+	OC: inh
Ceritinib	uk	uk
Cobimetinib	uk	uk
Crizotinib	+	uk
Dabrafenib	uk	uk
Dasatinib	+ (mice)	OC: inh
Entrectinib	uk	uk
Erdafitinib	uk	uk
Erlotinib	uk	OC: inh
Gefitinib	uk	OC: inh
Ibrutinib	uk	uk
Imatinib	±	OB: 0 OC: inh
Lapatinib	uk	OC: inh
Larotrectinib	uk	uk
Lenvatinib	uk	uk
Lorlatinib	uk	uk
Midostaurine	uk	OC: inh (mice)
Nilotinib	uk	OB: inh OC: inh
Osimertinib	uk	uk
Palbociclib	uk	uk
Pazopanib	uk	uk
Ponatinib	uk	uk
Pralsetinib	uk	uk
Regorafenib	uk	uk
Ribociclib	uk	uk
Ripretinib	uk	uk
Saracatinib	+	OB: 0 OC: inh
Sorafenib	uk	OC: inh
Sunitinib	- (mice)	OB: 0 OC: inh
Trametinib	uk	uk
Vandetanib	uk	OB: inh
Vemurafenib	uk	uk

*BMD* Bone mineral density, *OC* Osteoclastogenesis, *OB* osteoblast formation activity, *uk* Unknown, *inh* Inhibition, + indicates an increase of BMD,—indicates a decrease of BMD and 0 indicates an absence of effect

Globally, TKI treatments are well tolerated. The most frequent adverse events are digestive or hematological. Some bone adverse effects have been also described: periosteal reaction under pazopanib, osteonecrosis under sorafenib and sunitinib, secondary hyperparathyroidism under nilotinib, sunitinib and imatinib [102–108]. These adverse effects indicate that TKIs exert an effect on bone metabolism probably depending on their molecular target.

#### Effect on Bone Cells

Most TKIs inhibit osteoclastogenesis [100, 105, 109, 110]. This is not the case for afatinib [111]. Bosutinib, as a specific Src kinase inhibitor, has an action on osteoclast and osteoblast function. Interestingly, the c-kit inhibitory effect of bosutinib, is weak, making it a good alternative in children [112]. Bone resorption markers are also affected but differently in adults and children [100]. A decrease in markers in adults up to more than 50% in men has been noticed. Conversely, an increase in CTX in children on imatinib has been reported [113]. This inhibition is reversible or partially reversible upon discontinuation [100].

**Fig. 3:**
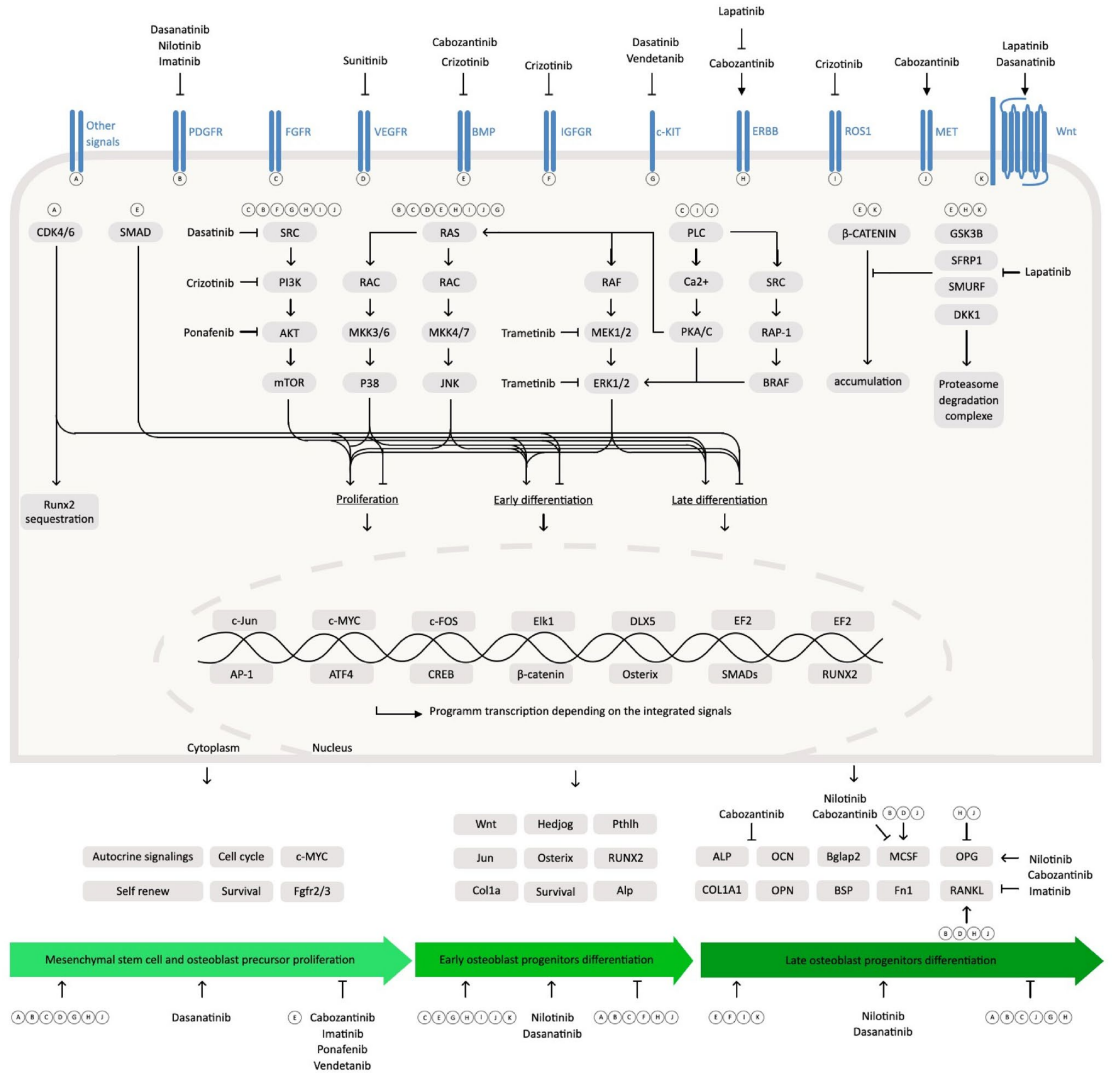
Integration of tyrosine kinase inhibitor (TKI) pathways in osteoblast signaling. Each membrane receptor may act on one or several intracellular pathway(s) resulting on biological effect(s) on osteoblast (OB) precursor proliferation, early and/or late OB differentiation. Intracellularly, TKI act mainly on PI3K/AKT/mTOR and MEK/ERK pathways

**Fig. 4:**
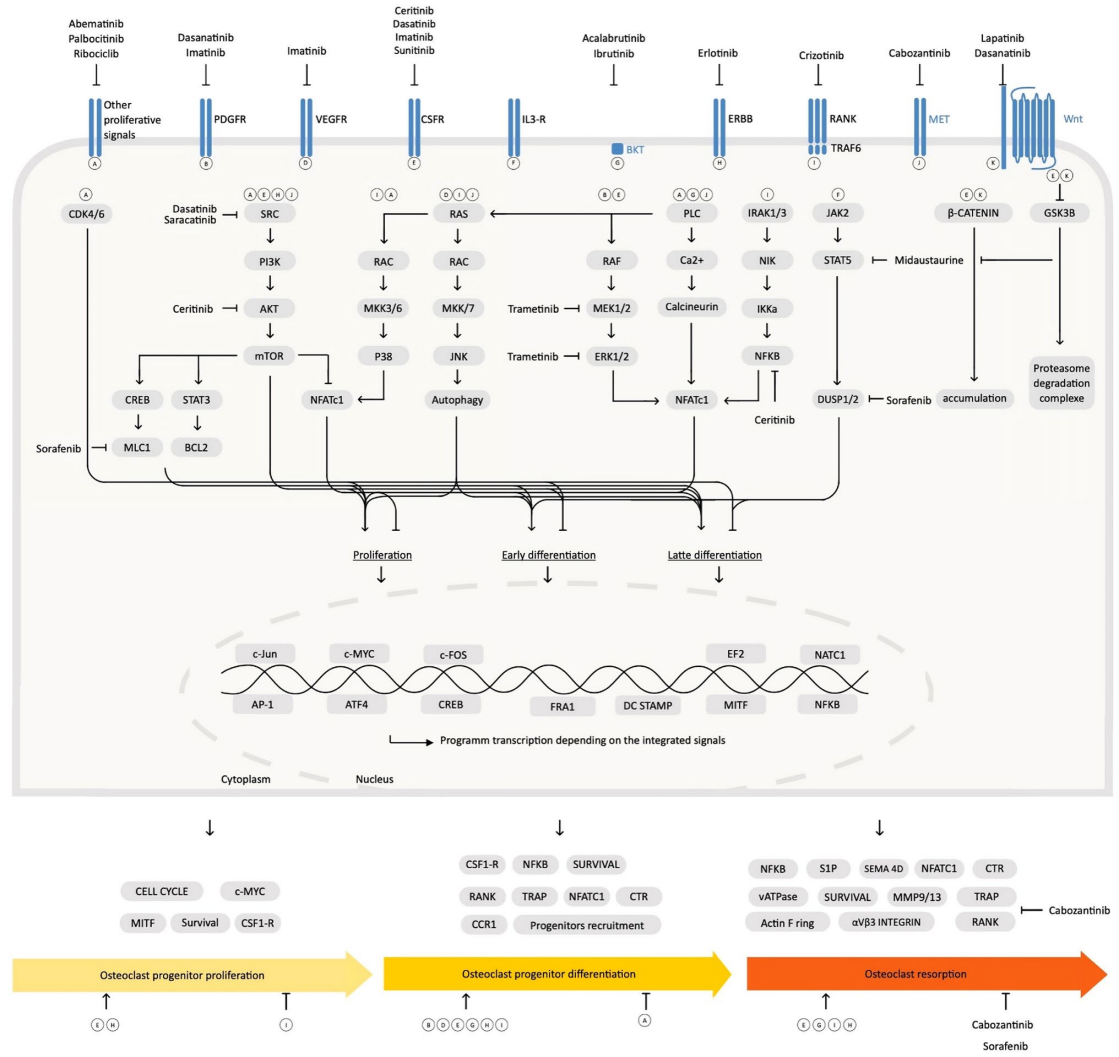
Integration of tyrosine kinase inhibitor (TKI) pathways in osteoclast signaling. Each membrane receptor may act on one or several intracellular pathway(s) resulting on biological effect(s) on osteoclast (OC) progenitor proliferation, OC progenitor differentiation and/or OC resorption activity

On the osteoblast, platelet-derived growth factor receptor (PDGFR) inhibition contributes to block osteoblast differentiation, proliferation, and mitogenesis in vitro [100, 101]. But in vivo, a large variability in results has been observed depending on the TKI. Saracatinib and bosutinib have no effect on bone formation markers [100, 112]. Even though a transient increase in Runx2 and osteocalcin at 3 months is observed on imatinib followed by a non-significant decrease at 18 months, this is dependent on the stage of osteoblast maturation [100, 105]. With nilotinib, the effect on osteoblasts may be neutral and accounts for the balance between c-abl and PDGFR activity [101]. Lastly, osteocalcin was shown decreased on vandetanib treatment [100]. These effects on bone remodeling can cause disproportionate effect or even destruction of cortical bone, bone demineralization, ischemia or increased trabecular volume or even growth retardation in children [100, 113–115]. The effects of each TKI on osteoclastogenesis and osteoblastogenesis are summarized in Table 2 and Fig. 3 and 4.

### Immune Checkpoint Inhibitors

Immune check point inhibitors aim at blocking the negative signal that prevents immune T-cells to activate and kill tumor cells. The use of this strategy started in the melanoma and lung cancer fields and is now widely proposed in adjuvant or in metastatic setting.

In a recent study, following the report of Moseley [116], Ye et al*.* reported a 20% increase of fracture incidence in patients on immune checkpoint inhibitors [117]. A possible explanation may come from the immune activation that would increase osteoclastogenesis and osteoclast activity and thus decrease bone mass.

In mice, the group of Johnson analyzed the bone phenotype of *PD-L1* knock out mice. They observed a decreased bone volume, an impaired bone micro-architecture and a decreased bone strength [118]. The same results have been observed during pharmacological blockade of PD1. Preliminary results support the immune hypothesis stating that an increase in T cells number and increased osteoclast activity alter PD1 blockade.

### mTor Inhibitors

mTor are kinases that regulate cell growth, angiogenesis and survival. Everolimus and temsirolimus are 2 mTor inhibitors. Everolimus is indicated, in combination, for the management of hormone receptor positive advanced breast cancer. Temsirolimus is indicated for the treatment of renal cell carcinoma and mantle cell lymphoma. The addition of everolimus to exemestane doubles the time to progression-free survival compared with exemestane alone in postmenopausal women with HER2 + breast cancer [119].

#### Effect on Fractures and BMD

Effects of mTor inhibitors on BMD in humans have not been studied. In mice, after 4 weeks of treatment, BMD improves and the bone volume/total volume ratio is 37% higher than in the control group [120]. There are no available fracture data.

#### Effect on Bone Cells

In vitro and on intervertebral disc cells, mTor inhibitors showed their anti-senescence and anti-apoptotic properties through anti-IL1β activity inhibition. They also exert an anti-catabolic effect (MMP-2,9,13 and TIMP-1, 2) on the bone matrix through the activation of signaling pathways involving the Akt family [121].

Bone marrow edema associated with fracture has been reported 6 months after initiation of everolimus. Resolution of the adverse effect was observed 2 weeks after discontinuation and appears to be correlated with a decrease in intraosseous perfusion. This event seems to be related to its action on bone metabolism since everolimus exerts a potential inhibitory effect on osteoclast formation and activity and on osteoblast differentiation by inhibiting osteoblast gene expression [122]. Browne et al*.* also show in vitro and in mice, that osteoclastic precursor viability and osteoclast differentiation markers decreased significantly under everolimus regardless of its concentration. At higher concentrations, markers of osteoblast activity (ALP, OCN, OPG and Runx2) were also decreased, but without affecting the viability of pre-osteoblasts. Interestingly, everolimus prevents the significant increase of bone turnover and bone loss induced by ovariectomy [120].

### Anti-Angiogenic Drugs

#### Anti-VEGF Antibodies

Vascular endothelial growth factor (VEGF) plays an essential role in vasculogenesis and angiogenesis. Levels of VEGF are increased in the serum of patients with primary malignant bone tumors [123, 124]. Bevacizumab binds to VEGF, inhibiting the binding of VEGF to its receptors on the surface of endothelial cells. At the tumor level, bevacizumab inhibits the formation of new tumor vessels and tumor growth.

No data are available on the risk of fracture and BMD with bevacizumab. However, the combination of a VEGF inhibitor and a VEGF receptor inhibitor is superior to VEGF inhibitor therapy alone on the progression of bone metastases in animals [125]. Moreover, anti-angiogenic drugs are known to induce osteonecrosis, particularly when combined with bisphosphonates (> 10%). However, osteonecrosis of the jaw has been observed without associated risk factors [126, 127]. These events are dose- and time-dependent and can be explained by their mechanism of action. In rabbits, VEGF regulates osteoclast differentiation and stimulates bone resorption [128].

#### Thalidomide and its Derivatives

Thalidomide, lenalidomide and pomalidomide are immunomodulators (IMIDs) used in the treatment of myeloma or lymphoma. These drugs inhibit angiogenesis and belong to the class of anti-angiogenics. However, there are no studies describing the effects of anti-angiogenics on BMD and fracture risk.

Thalidomide and lenalidomide have a significant inhibitory effect on osteoblast differentiation, even at the lowest doses [129]. This inhibition is observed at the late stages of differentiation, accompanied by a reduction in matrix mineralization and a decrease in ALP. In addition, the expression of the bone-forming transcription factors *Runx2* and *DLX-5* is decreased while the expression of the inhibitory proteins DKK-1 and inhibin βA is increased. In *vitro*, pomalidomide totally inhibits RANKL-induced osteoclast formation over a 21-day test period. This inhibition is observed early in the differentiation stages of hematopoietic progenitor cells, also resulting from a negative regulation of the transcription factor PU.1. These data are confirmed by a significant decrease in CFU-GM precursors without any toxic effect [130].

These in vitro data were confirmed in vivo [130]. Lenalidomide and pomalidomide directly inhibit osteoclasts through the inhibition of αVβ3-integrin and cathepsin K expression and indirectly by decreasing in myeloma cells, protein expression levels of RANKL (but not OPG) conducting to a decrease in RANKL/OPG ratio and by limiting APRIL, BAFF and MIP-1α [131, 132]. Immunomodulating drugs thus exert a direct and indirect inhibitory effect on osteoclasts.

### Intravenous Chemotherapy

Anti-cancer management also relies on cytostatics, which are not targeted therapies. For some of them, the effects on bone or BMD are described. Most of the anti-cancer drugs listed are used in neoadjuvant chemotherapy of primary malignant bone tumors (methotrexate, cyclophosphamide, doxorubicin, ifosfamide and 5-fluorouracil), such as primary malignant osteosarcoma and Ewing´s family of tumors [133, 134]. These tumors predominantly affect children or adolescents during accrual of peak bone mass which may be disrupted by the treatment. Animal studies show that chemotherapy has a profound negative effect on all measures of bone mass and cortical and cancellous bone architecture [135]. While some drugs appear to significantly reduce BMD, it has not yet been conclusively established that this reduced BMD is correlated with an increased fracture risk [136, 137].

#### 5-Fluorouracil (5-FU)

In vitro, both aspects of osteoclast physiology decrease under 5-FU: differentiation in a dose-dependent manner, and activation as assessed by MCP-1, IL-1β, and VEGF production. In mice, the expression of genes involved in osteoclastogenesis is inhibited via the NF-κB signaling pathway [138].

#### Cyclophosphamide, Methotrexate, Doxorubicin, Ifosfamide

*Effect on BMD.* In the treatment of breast cancer in premenopausal women, after 1 year of amenorrhea, the decrease in BMD is about 6% in the spine and 5% in the femur under cyclophosphamide and methotrexate [25]. In children, the cumulative dose of methotrexate > 40,000 mg/m^2^ is associated with a reduction in BMD with a decrease in bone volume and an increased risk of osteopenia [1, 136, 137]. In the long term, ifosfamide causes a decrease in BMD in children [1, 136, 137].

*Effect on bone.* Methotrexate exerts a cytotoxic effect on the proliferation of pre-osteoblasts and on the osteoblasts with an alteration of bone formation and in parallel to a stimulation of osteoclast activity [1]. These same effects are found in vitro under doxorubicin [1, 12].

## Conclusion

Beyond glucocorticoid and hormone therapies, the bone effects of anticancer drugs are only partially evaluated. Since overall survival and recurrence-free survival have been drastically improved in many cancers, the impact of these drugs on bone cells, bone density and fracture risk is a major issue to investigate in order to improve patients’ long-term quality of life during the “post-cancer time”. Figure 2.

In addition, to the bone effects of these drugs, physicians should be aware that these drugs may also have distinct effects on muscle which may contribute to an increased risk of falls. Furthermore, tumor-related osteosarcopenia and cachexia may have a further negative impact on bone health in these patients.

Therefore altogether the oncologist, the bone metabolism specialist, and the general practitioners need to be aware of the effects of oncology treatments on the skeleton. In the future, clinical trials documenting efficacy of osteoporosis therapies in protecting from adverse skeletal outcomes need to be performed. This is one of the numerous upcoming challenges for cancer and bone physicians alike.

## Supplementary Information

Below is the link to the electronic supplementary material.

Supplementary file1 (DOCX 2897 KB)

## References

[1] Marcucci G, Beltrami G, Tamburini A, Body JJ, Confavreux CB, Hadji P et al (2019) Bone health in childhood cancer: review of the literature and recommendations for the management of bone health in childhood cancer survivors. Ann Oncol 30(6):908–920 31111878 10.1093/annonc/mdz120

[2] Wilson CL, Ness KK (2013) Bone mineral density deficits and fractures in survivors of childhood cancer. Curr Osteoporos Rep 11(4):329–337 24043370 10.1007/s11914-013-0165-0 PMC4260527

[3] Confavreux CB, Follet H, Mitton D, Pialat JB, Clézardin P (2021) Fracture Risk Evaluation of Bone Metastases: A Burning Issue. Cancers (Basel) 13(22):5711 34830865 10.3390/cancers13225711 PMC8616502

[4] Rizzoli R, Biver E (2015) Glucocorticoid-induced osteoporosis: who to treat with what agent? Nat Rev Rheumatol 11(2):98–109 25385412 10.1038/nrrheum.2014.188

[5] Subbu K, Renner JB, Rubin JE (2023) Extensive osteonecrosis after glucocorticoids: clinical response to bisphosphonate. JCEM Case Rep 1(1):luac006 37908238 10.1210/jcemcr/luac006 PMC10578391

[6] Canalis E, Mazziotti G, Giustina A, Bilezikian JP (2007) Glucocorticoid-induced osteoporosis: pathophysiology and therapy. Osteoporos Int 18(10):1319–1328 17566815 10.1007/s00198-007-0394-0

[7] Van Staa T, Leukfens H, Cooper C (2002) The epidemiology of corticosteroid-induced osteoporosis: a meta-analysis. Osteoporos Int 70(13):777–787 10.1007/s001980200108 12378366

[8] Van Staa TP, Leufkens HGM, Abenhaim L, Zhang B, Cooper C (2000) Use of oral corticosteroids and risk of fractures. J Bone Miner Res 15(6):993–1000 10841167 10.1359/jbmr.2000.15.6.993

[9] Kanis JA, Johansson H, Oden A, Johnell O, de Laet C, Melton LJ III et al (2004) A meta-analysis of prior corticosteroid use and fracture risk. J Bone Miner Res 19(6):893–899 15125788 10.1359/JBMR.040134

[10] Velentza L, Zaman F, Sävendahl L (2021) Bone health in glucocorticoid-treated childhood acute lymphoblastic leukemia. Crit Rev Oncol/Hematol 168:103492 34655742 10.1016/j.critrevonc.2021.103492

[11] Leonard MB, Feldman HI, Shults J, Zemel BS, Foster BJ, Stallings VA (2004) Long-term, high-dose glucocorticoids and bone mineral content in childhood glucocorticoid-sensitive nephrotic syndrome. N Engl J Med 351(9):868–875 15329424 10.1056/NEJMoa040367

[12] Wasilewski-Masker K, Kaste SC, Hudson MM, Esiashvili N, Mattano LA, Meacham LR (2008) Bone mineral density deficits in survivors of childhood cancer: long-term follow-up guidelines and review of the literature. Pediatrics 121(3):e705–e713 18310191 10.1542/peds.2007-1396

[13] Beier EE, Sheu TJ, Resseguie EA, Takahata M, Awad HA, Cory-Slechta DA et al (2017) Sclerostin activity plays a key role in the negative effect of glucocorticoid signaling on osteoblast function in mice. Bone Res 5:17013 28529816 10.1038/boneres.2017.13 PMC5422922

[14] Ding H, Wang T, Xu D, Cha B, Liu J, Li Y (2015) Dexamethasone-induced apoptosis of osteocytic and osteoblastic cells is mediated by TAK1 activation. Biochem Biophys Res Commun 460(2):157–163 25753204 10.1016/j.bbrc.2015.02.161

[15] Yager JD, Davidson NE (2006) Estrogen carcinogenesis in breast cancer. N Engl J Med 354(3):270–282 16421368 10.1056/NEJMra050776

[16] Kurebayashi J, Shiba E, Toyama T, Matsumoto H, Okazaki M, Nomizu T (2021) A follow-up study of a randomized controlled study evaluating safety and efficacy of leuprorelin acetate every-3-month depot for 2 versus 3 or more years with tamoxifen for 5 years as adjuvant treatment in premenopausal patients with endocrine-responsive breast cancer. Breast Cancer 28(3):684–697 33638810 10.1007/s12282-020-01205-w PMC8064970

[17] Lambertini M, Boni L, Michelotti A, Magnolfi E, Cogoni AA, Mosconi AM et al (2021) Long-term outcomes with pharmacological ovarian suppression during chemotherapy in premenopausal early breast cancer patients. J Natl Cancer Inst 114(3):400–408 10.1093/jnci/djab213 PMC8902441 34850043

[18] Ziaran S, Goncalves FM, Sn JB (2013) Complex metabolic and skeletal changes in men taking long-term androgen deprivation therapy. Clin Genitourin Cancer 11(1):33–38 23000203 10.1016/j.clgc.2012.08.005

[19] Abufaraj M, Iwata T, Kimura S, Haddad A, Al-Ani H, Abusubaih L et al (2021) Differential impact of gonadotropin-releasing hormone antagonist versus agonist on clinical safety and oncologic outcomes on patients with metastatic prostate cancer: a meta-analysis of randomized controlled trials. Eur Urol 79(1):44–53 32605859 10.1016/j.eururo.2020.06.002

[20] Briot K, Paccou J, Beuzeboc P, Bonneterre J, Bouvard B, Confavreux CB et al (2019) French recommendations for osteoporosis prevention and treatment in patients with prostate cancer treated by androgen deprivation. Joint Bone Spine 86(1):21–28 30287350 10.1016/j.jbspin.2018.09.017

[21] Shahinian VB, Kuo YF, Freeman JL, Goodwin JS (2005) Risk of fracture after androgen deprivation for prostate cancer. N Engl J Med 352(2):154–164 15647578 10.1056/NEJMoa041943

[22] Hatano T, Oishi Y, Furuta A, Iwamuro S, Tashiro K (2000) Incidence of bone fracture in patients receiving luteinizing hormone-releasing hormone agonists for prostate cancer. BJU Int 86(4):449–452 10971270 10.1046/j.1464-410x.2000.00774.x

[23] Mohamad NV, Ima-Nirwana S, Chin KY (2023) The skeletal effects of gonadotropin-releasing hormone antagonists: a concise review. Endocr, Metab Immune Disord - Drug Targets 21(10):1713–1720 10.2174/1871530321666201216164410 33327926

[24] Ramchand SK, Cheung YM, Yeo B, Grossmann M (2019) The effects of adjuvant endocrine therapy on bone health in women with breast cancer. J Endocrinol 241(3):R111–R124 30991355 10.1530/JOE-19-0077

[25] Bouvard B, Confavreux CB, Briot K, Bonneterre J, Cormier C, Cortet B et al (2019) French recommendations on strategies for preventing and treating osteoporosis induced by adjuvant breast cancer therapies. Joint Bone Spine 86(5):542–553 31352137 10.1016/j.jbspin.2019.07.005

[26] Eastell R, Adams J, Clack G, Howell A, Cuzick J, Mackey J et al (2011) Long-term effects of anastrozole on bone mineral density: 7-year results from the ATAC trial. Ann Oncol 22:857–862 20929964 10.1093/annonc/mdq541

[27] Miyaji Y, Saika T, Yamamoto Y, Kusaka N, Arata R, Ebara S et al (2004) Effects of gonadotropin-releasing hormone agonists on bone metabolism markers and bone mineral density in patients with prostate cancer. Urology 64(1):128–131 15245949 10.1016/j.urology.2004.03.012

[28] Smith MR, Fallon MA, Goode MJ (2003) Cross-sectional study of bone turnover during bicalutamide monotherapy for prostate cancer. Urology 61(1):127–131 12559282 10.1016/s0090-4295(02)02006-x

[29] Bargiota A, Oeconomou A, Zachos I, Samarinas M, Pisters LL, Tzortzis V (2020) Adverse effects of androgen deprivation therapy in patients with prostate cancer: focus on muscle and bone health. J BUON 25(3):1286–1294 32862568

[30] Chi KN, Agarwal N, Bjartell A, Chung BH, de Santana P, Gomes AJ, Given R et al (2019) Apalutamide for metastatic, castration-sensitive prostate cancer. N Engl J Med 381(1):13–24 31150574 10.1056/NEJMoa1903307

[31] Armstrong AJ, Szmulewitz RZ, Petrylak DP, Holzbeierlein J, Villers A, Azad A et al (2019) ARCHES: a randomized, phase iii study of androgen deprivation therapy with enzalutamide or placebo in men with metastatic hormone-sensitive prostate cancer. J Clin Oncol 37(32):2974–2986 31329516 10.1200/JCO.19.00799 PMC6839905

[32] Bouleftour W, Boussoualim K, Sotton S, Vassal C, Thomas T, Magne N et al (2021) Second-generation hormonotherapy in prostate cancer and bone microenvironment. Endocr.-Related Cancer 28(8):T39-49 10.1530/ERC-21-0118 33974558

[33] Fizazi K, Shore N, Tammela TL, Ulys A, Vjaters E, Polyakov S et al (2019) Darolutamide in nonmetastatic, castration-resistant prostate cancer. N Engl J Med 380(13):1235–1246 30763142 10.1056/NEJMoa1815671

[34] Wu CC, Chen PY, Wang SW, Tsai MH, Wang YCL, Tai CL et al (2021) Risk of fracture during androgen deprivation therapy among patients with prostate cancer: a systematic review and meta-analysis of cohort studies. Front Pharmacol 12:652979 34421586 10.3389/fphar.2021.652979 PMC8378175

[35] Spry A, Galvão S, Davies R, La Bianca S, Joseph D, Davdison A (2009) Long-term effects of intermittent androgen suppression on testosterone recovery and bone mineral density: results of a 33-months observation study. BJU Int 6(104):806–812 10.1111/j.1464-410X.2009.08458.x 19281463

[36] Formenti AM, Dalla Volta A, di Filippo L, Berruti A, Giustina A (2021) Effects of medical treatment of prostate cancer on bone health. Trends Endocrinol & Metab 32(3):135–158 33509658 10.1016/j.tem.2020.12.004

[37] Preston DM, Torréns JI, Harding P, Howard RS, Duncan WE, McLeod DG (2002) Androgen deprivation in men with prostate cancer is associated with an increased rate of bone loss. Prostate Cancer Prostatic Dis 5(4):304–310 12627216 10.1038/sj.pcan.4500599

[38] Wadhwa VK, Weston R, Parr NJ (2011) Bicalutamide monotherapy preserves bone mineral density, muscle strength and has significant health-related quality of life benefits for osteoporotic men with prostate cancer. BJU Int 107(12):1923–1929 20950306 10.1111/j.1464-410X.2010.09726.x

[39] Su S, Cao J, Meng X, Liu R, Vander Ark A, Woodford E et al (2022) Enzalutamide-induced and PTH1R-mediated TGFBR2 degradation in osteoblasts confers resistance in prostate cancer bone metastases. Cancer Lett 525:170–178 34752846 10.1016/j.canlet.2021.10.042 PMC9669895

[40] Fisher B, Costantino JP, Wickerham DL, Cecchini RS, Cronin WM, Robidoux A et al (2005) Tamoxifen for the prevention of breast cancer: current status of the national surgical adjuvant breast and bowel project P-1 study. JNCI: J National Cancer Inst 97(22):1652–1662 10.1093/jnci/dji372 16288118

[41] Vestergaard P, Rejnmark L, Mosekilde L (2008) Effect of tamoxifen and aromatase inhibitors on the risk of fractures in women with breast cancer. Calcif Tissue Int 82(5):334–340 18463912 10.1007/s00223-008-9132-7

[42] Eastell R, Hannon RA, Cuzick J, Dowsett M, Clack G, Adams JE et al (2006) Effect of an aromatase inhibitor on bmd and bone turnover markers: 2-year results of the Anastrozole, Tamoxifen, Alone or in Combination (ATAC) trial (18233230). J Bone Miner Res 21:1215–1223 16869719 10.1359/jbmr.060508

[43] Kyvernitakis I, Kostev K, Hadji P (2018) The tamoxifen paradox-influence of adjuvant tamoxifen on fracture risk in pre- and postmenopausal women with breast cancer. Osteoporos Int 29(11):2557–2564 30032359 10.1007/s00198-018-4642-2

[44] Love RR, Mazess RB, Barden HS, Epstein S, Newcomb PA, Jordan VC et al (1992) Effects of tamoxifen on bone mineral density in postmenopausal women with breast cancer. N Engl J Med 26(326):852–856 10.1056/NEJM199203263261302 1542321

[45] Powles TJ, Hickish T, Kanis JA, Tidy A, Ashley S (1996) Effect of tamoxifen on bone mineral density measured by dual-energy x-ray absorptiometry in healthy premenopausal and postmenopausal women. J Clin Oncol 14:78–84 8558225 10.1200/JCO.1996.14.1.78

[46] Maximov PY, Lee TM, Jordan VC (2013) The discovery and development of selective estrogen receptor modulators (SERMs) for clinical practice. Curr Clin Pharmacol 8(2):135–155 23062036 10.2174/1574884711308020006 PMC3624793

[47] Sverrisdottir A, Fornander T, Jacobsson H, von Schoultz E, Rutqvist LE (2004) Bone mineral density among premenopausal women with early breast cancer in a randomized trial of adjuvant endocrine therapy. J Clin Oncol 22:3694–3699 15365065 10.1200/JCO.2004.08.148

[48] Hadji P, Ziller M, Kieback D, Menschik T, Kalder M, Kuck J (2009) The effect of exemestane or tamoxifen on markers on bone turnover : results of a German sub-study of the tamoxifen exemestane adjuvant multicentre (TEAM) trial. Breast 3(18):159–164 10.1016/j.breast.2009.03.003 19364653

[49] Aihara T, Suemasu K, Takei H, Hozumi Y, Takehara M, Saito T et al (2010) Effects of exemestane, anastrozole and tamoxifen on bone mineral density and bone turnover markers in postmenopausal early breast cancer patients: results of N-SAS BC 04, the TEAM Japan substudy. Oncology 79:376–381 21430407 10.1159/000323489

[50] McCloskey EV, Hannon RA, Lakner G, Fraser WD, Clack G, Miyamoto A et al (2007) Effects of third generation aromatase inhibitors on bone health and other safety parameters: results of an open, randomised, multi-centre study of letrozole, exemestane and anastrozole in healthy postmenopausal women. Eur J Cancer 43:2523–2531 18029171 10.1016/j.ejca.2007.08.029

[51] Howell A, Cuzick J, Baum M, Buzdar A, Dowsett M, Forbes JF et al (2005) Results of the ATAC (Arimidex, Tamoxifen, Alone or in Combination) trial after completion of 5 years’ adjuvant treatment for breast cancer. Lancet 365:60–62 15639680 10.1016/S0140-6736(04)17666-6

[52] Coleman RE, Banks LM, Girgis SI, Kilburn LS, Vrdoljak E, Fox J et al (2007) Skeletal effects of exemestane on bone-mineral density, bone biomarkers, and fracture incidence in postmenopausal women with early breast cancer participating in the Intergroup Exemestane Study (IES): a randomised controlled study. Lancet Oncol 8(2):119–127 17267326 10.1016/S1470-2045(07)70003-7

[53] Rabaglio M, Sun Z, Price KN, Castiglione-Gertsch M, Hawle H, Thurlimann B et al (2009) Bone fractures among postmenopausal patients with endocrine-responsive early breast cancer treated with 5 years of letrozole or tamoxifen in the BIG 1–98 trial. Ann Oncol 20:1489–1498 19474112 10.1093/annonc/mdp033 PMC2731016

[54] Jakesz R, Jonat W, Gnant M, Mittlboeck M, Greil R, Tausch C et al (2005) Switching of postmenopausal women with endocrine-responsive early breast cancer to anastrozole after 2 years’ adjuvant tamoxifen: combined results of ABCSG trial 8 and ARNO 95 trial. Lancet 366:455–462 16084253 10.1016/S0140-6736(05)67059-6

[55] Arimidex, Tamoxifen, Alone or in Combination Trialists’ Group, Buzdar A, Howell A, Cuzick J, Wale C, Distler W, et al. (2006). Comprehensive side-effect profile of anastrozole and tamoxifen as adjuvant treatment for early-stage breast cancer: long-term safety analysis of the ATAC trial. Lancet Oncol. 7(8): 633‑43. 10.1016/S1470-2045(06)70767-7 16887480

[56] Coates AS, Keshaviah A, Thürlimann B, Mouridsen H, Mauriac L, Forbes JF et al (2007) Five years of letrozole compared with tamoxifen as initial adjuvant therapy for postmenopausal women with endocrine-responsive early breast cancer: update of study BIG 1–98. J Clin Oncol 25(5):486–492 17200148 10.1200/JCO.2006.08.8617

[57] Jakesz R, Greil R, Gnant M, Schmid M, Kwasny W, Kubista E et al (2007) Extended adjuvant therapy with anastrozole among postmenopausal breast cancer patients: results from the randomized Austrian breast and colorectal cancer study group trial 6a. J Natl Cancer Inst 99:1845–1853 18073378 10.1093/jnci/djm246

[58] Coombes RC, Kilburn LS, Snowdon CF, Paridaens R, Coleman RE, Jones SE et al (2007) Survival and safety of exemestane versus tamoxifen after 2–3 years’ tamoxifen treatment (Intergroup Exemestane Study): a randomised controlled trial. Lancet 369(9561):559–570 17307102 10.1016/S0140-6736(07)60200-1

[59] Goss PE, Ingle JN, Martino S, Robert NJ, Muss HB, Piccart MJ et al (2005) Randomized trial of letrozole following tamoxifen as extended adjuvant therapy in receptor-positive breast cancer: updated findings from NCIC CTG MA.17. J Natl Cancer Inst 97:1262–1271 16145047 10.1093/jnci/dji250

[60] Colzani E, Clements M, Johansson ALV, Liljegren A, He W, Brand J et al (2016) Risk of hospitalisation and death due to bone fractures after breast cancer: a registry-based cohort study. Br J Cancer 115(11):1400–1407 27701383 10.1038/bjc.2016.314 PMC5129831

[61] Arimidex TA or in CTG, Forbes JF, Cuzick J, Buzdar A, Howell A, Tobias JS, et al. (2008) Effect of anastrozole and tamoxifen as adjuvant treatment for early-stage breast cancer: 100-month analysis of the ATAC trial. Lancet Oncol. 9: 45‑53. 10.1016/S1470-2045(07)70385-6 18083636

[62] Jerzak KJ, Raphael J, Desautels DN, Blanchette PS, Tyono I, Pritchard KI (2018) Bone-targeted therapy in early breast cancer. Oncology (Williston Park) 32(11):562–569 30474104

[63] Goss PE, Hershman DL, Cheung AM, Ingle JN, Khosla S, Stearns V et al (2014) Effects of adjuvant exemestane versus anastrozole on bone mineral density for women with early breast cancer (MA.27B): a companion analysis of a randomised controlled trial. Lancet Oncol 15:474–482 24636210 10.1016/S1470-2045(14)70035-X PMC4352316

[64] Goldvaser H, Barnes TA, Šeruga B, Cescon DW, Ocaña A, Ribnikar D et al (2018) Toxicity of extended adjuvant therapy with aromatase inhibitors in early breast cancer: a systematic review and meta-analysis. J Natl Cancer Inst. 10.1093/jnci/djx141 28922781 10.1093/jnci/djx141

[65] Perez EA, Josse RG, Pritchard KI, Ingle JN, Martino S, Findlay BP et al (2006) Effect of letrozole versus placebo on bone mineral density in women with primary breast cancer completing 5 or more years of adjuvant tamoxifen: a companion study to NCIC CTG MA.17. J Clin Oncol 24:3629–3635 16822845 10.1200/JCO.2005.05.4882

[66] Decensi A, Sun Z, Guerrieri-Gonzaga A, Thurlimann B, McIntosh C, Tondini C et al (2014) Bone mineral density and circulating biomarkers in the BIG 1–98 trial comparing adjuvant letrozole, tamoxifen and their sequences. Breast Cancer Res Treat 144:321–329 24487691 10.1007/s10549-014-2849-2 PMC3982877

[67] Confavreux CB, Fontana A, Guastalla JP, Munoz F, Brun J, Delmas PD (2007) Estrogen-dependent increase in bone turnover and bone loss in postmenopausal women with breast cancer treated with anastrozole. Prev Bisphosphonates Bone 41(3):346–352 10.1016/j.bone.2007.06.004 17618847

[68] Eastell R, Adams J, Coleman R, Howell A, Hannon R, Cuzick J (2008) Effect of anastrozole on bone mineral density: 5-years results from the anastrozole, tamoxifen, alone or in combination trial 18233230. J Clin Oncol 7(26):1051–1057 10.1200/JCO.2007.11.0726 18309940

[69] Goss PE, Ingle JN, Pritchard KI, Ellis MJ, Sledge GW, Budd GT et al (2013) Exemestane versus anastrozole in postmenopausal women with early breast cancer: NCIC CTG MA.27–a randomized controlled phase III trial. J Clin Oncol 31:1398–1404 23358971 10.1200/JCO.2012.44.7805 PMC3612593

[70] Goss PE, Qi S, Josse RG, Pritzker KP, Mendes M, Hu H et al (2004) The steroidal aromatase inhibitor exemestane prevents bone loss in ovariectomized rats. Bone 34:384–392 15003786 10.1016/j.bone.2003.11.006

[71] Cheung AM, Tile L, Cardew S, Pruthi S, Robbins J, Tomlinson G et al (2012) Bone density and structure in healthy postmenopausal women treated with exemestane for the primary prevention of breast cancer: a nested substudy of the MAP.3 randomised controlled trial. Lancet Oncol 13:275–284 22318095 10.1016/S1470-2045(11)70389-8

[72] Rizzoli R, Body JJ, DeCensi A, De Censi A, Reginster JY, Piscitelli P et al (2012) Guidance for the prevention of bone loss and fractures in postmenopausal women treated with aromatase inhibitors for breast cancer: an ESCEO position paper. Osteoporos Int 23(11):2567–2576 22270857 10.1007/s00198-011-1870-0

[73] Su G, Xiang Y, He G, Jiang C, Li C, Yan Z et al (2014) Bisphosphonates may protect against bone loss in postmenopausal women with early breast cancer receiving adjuvant aromatase inhibitor therapy: results from a meta-analysis. Arch Med Res 45(7):570–579 25450582 10.1016/j.arcmed.2014.10.007

[74] Kyvernitakis I, Kann PH, Thomasius F, Hars O, Hadji P (2018) Prevention of breast cancer treatment-induced bone loss in premenopausal women treated with zoledronic acid: final 5-year results from the randomized, double-blind, placebo-controlled ProBONE II trial. Bone 114:109–115 29908297 10.1016/j.bone.2018.06.007

[75] Gnant MF, Mlineritsch B, Luschin-Ebengreuth G, Grampp S, Kaessmann H, Schmid M et al (2007) Zoledronic acid prevents cancer treatment-induced bone loss in premenopausal women receiving adjuvant endocrine therapy for hormone-responsive breast cancer: a report from the Austrian breast and colorectal cancer study group. J Clin Oncol 25:820–828 17159195 10.1200/JCO.2005.02.7102

[76] Coleman R, de Boer R, Eidtmann H, Llombart A, Davidson N, Neven P et al (2013) Zoledronic acid (zoledronate) for postmenopausal women with early breast cancer receiving adjuvant letrozole (ZO-FAST study): final 60-month results. Ann Oncol 24(2):398–405 23047045 10.1093/annonc/mds277

[77] Brufsky AM, Harker WG, Beck JT, Bosserman L, Vogel C, Seidler C et al (2012) Final 5-year results of Z-FAST trial: adjuvant zoledronic acid maintains bone mass in postmenopausal breast cancer patients receiving letrozole. Cancer 118(5):1192–1201 21987386 10.1002/cncr.26313

[78] Ellis GK, Bone HG, Chlebowski R, Paul D, Spadafora S, Smith J et al (2008) Randomized trial of denosumab in patients receiving adjuvant aromatase inhibitors for nonmetastatic breast cancer. J Clin Oncol 26(30):4875–4882 18725648 10.1200/JCO.2008.16.3832

[79] Ellis GK, Bone HG, Chlebowski R, Paul D, Spadafora S, Fan M et al (2009) Effect of denosumab on bone mineral density in women receiving adjuvant aromatase inhibitors for non-metastatic breast cancer: subgroup analyses of a phase 3 study. Breast Cancer Res Treat 118(1):81–87 19308727 10.1007/s10549-009-0352-y

[80] Gnant M, Pfeiler G, Dubsky PC, Hubalek M, Greil R, Jakesz R et al (2015) Adjuvant denosumab in breast cancer (ABCSG-18): a multicentre, randomised, double-blind, placebo-controlled trial. Lancet 386:433–443 26040499 10.1016/S0140-6736(15)60995-3

[81] Early Breast Cancer Trialists’ Collaborative Group (EBCTCG) (2015) Adjuvant bisphosphonate treatment in early breast cancer: meta-analyses of individual patient data from randomised trials. Lancet 386(10001):1353–1361 26211824 10.1016/S0140-6736(15)60908-4

[82] Smith MR, Eastham J, Gleason DM, Shasha D, Tchekmedyian S, Zinner N (2003) Randomized controlled trial of zoledronic acid to prevent bone loss in men receiving androgen deprivation therapy for nonmetastatic prostate cancer. J Urol 169(6):2008–2012 12771706 10.1097/01.ju.0000063820.94994.95

[83] Ryan CW, Huo D, Demers LM, Beer TM, Lacerna LV (2006) Zoledronic acid initiated during the first year of androgen deprivation therapy increases bone mineral density in patients with prostate cancer. J Urol 176(3):972–978 16890673 10.1016/j.juro.2006.04.078

[84] Michaelson MD, Kaufman DS, Lee H, McGovern FJ, Kantoff PW, Fallon MA et al (2007) Randomized controlled trial of annual zoledronic acid to prevent gonadotropin-releasing hormone agonist-induced bone loss in men with prostate cancer. J Clin Oncol 25(9):1038–1042 17369566 10.1200/JCO.2006.07.3361 PMC3047397

[85] Bhoopalam N, Campbell SC, Moritz T, Broderick WR, Iyer P, Arcenas AG et al (2009) Intravenous zoledronic acid to prevent osteoporosis in a veteran population with multiple risk factors for bone loss on androgen deprivation therapy. J Urol 182(5):2257–2264 19758618 10.1016/j.juro.2009.07.046

[86] Greenspan SL, Nelson JB, Trump DL, Resnick NM (2007) Effect of once-weekly oral alendronate on bone loss in men receiving androgen deprivation therapy for prostate cancer: a randomized trial. Ann Intern Med 146(6):416–424 17371886 10.7326/0003-4819-146-6-200703200-00006

[87] Planas J, Trilla E, Raventós C, Cecchini L, Orsola A, Salvador C et al (2009) Alendronate decreases the fracture risk in patients with prostate cancer on androgen-deprivation therapy and with severe osteopenia or osteoporosis. BJU Int 104(11):1637–1640 19549260 10.1111/j.1464-410X.2009.08622.x

[88] Klotz LH, McNeill IY, Kebabdjian M, Zhang L, Chin JL, Canadian Urology Research Consortium. (2013). A phase 3, double-blind, randomised, parallel-group, placebo-controlled study of oral weekly alendronate for the prevention of androgen deprivation bone loss in nonmetastatic prostate cancer: the Cancer and Osteoporosis Research with Alendronate and Leuprolide (CORAL) study. Eur Urol. 63(5):927‑35. 10.1016/j.eururo.2012.09.007 23040208

[89] Coleman R, Hadji P, Body JJ, Santini D, Chow E, Terpos E et al (2020) Bone health in cancer: ESMO clinical practice guidelines†. Ann Oncol 31(12):1650–1663 32801018 10.1016/j.annonc.2020.07.019

[90] Scott K, Hayden PJ, Will A, Wheatley K, Coyne I (2016) Bortezomib for the treatment of multiple myeloma. Cochrane Database Systematic Rev [Internet]. 10.1002/14651858.CD010816.pub2 10.1002/14651858.CD010816.pub2 PMC10387344 27096326

[91] Li SJ, Hao J, Mao Y, Si YL (2020) Effects of the proteasome inhibitor bortezomib in combination with chemotherapy for the treatment of mantle cell lymphoma: a meta-analysis. Turk J Haematol 37(1):13–19 31464119 10.4274/tjh.galenos.2019.2019.0128 PMC7057744

[92] Xu W, Sun X, Wang B, Guo H (2018) Pooled analysis of the reports of carfilzomib/ixazomib combinations for relapsed/refractory multiple myeloma. Ann Hematol 97(2):299–307 29159498 10.1007/s00277-017-3173-9

[93] Wang H, Zhang H, Srinivasan V, Tao J, Sun W, Lin X et al (2020) Targeting bortezomib to bone increases its bone anabolic activity and reduces systemic adverse effects in mice. J Bone Miner Res 35(2):343–356 31610066 10.1002/jbmr.3889 PMC10587833

[94] Accardi F, Toscani D, Bolzoni M, Dalla Palma B, Aversa F, Giuliani N (2015) Mechanism of action of bortezomib and the new proteasome inhibitors on myeloma cells and the bone microenvironment: impact on myeloma-induced alterations of bone remodeling. Biomed Res Int 2015:172458 26579531 10.1155/2015/172458 PMC4633537

[95] Zangari M, Suva LJ (2016) The effects of proteasome inhibitors on bone remodeling in multiple myeloma. Bone 86:131–138 26947893 10.1016/j.bone.2016.02.019 PMC5516941

[96] Tibullo D, Longo A, Vicario N, Romano A, Barbato A, Di Rosa M et al (2020) Ixazomib improves bone remodeling and counteracts sonic hedgehog signaling inhibition mediated by myeloma cells. Cancers (Basel) 12(2):323 32019102 10.3390/cancers12020323 PMC7073172

[97] Rath S, Elamarthi P, Parab P, Gulia S, Nandhana R, Mokal S et al (2021) Efficacy and safety of palbociclib and ribociclib in patients with estrogen and/or progesterone receptor positive, HER2 receptor negative metastatic breast cancer in routine clinical practice. PLoS ONE 16(7):e0253722 34292933 10.1371/journal.pone.0253722 PMC8297817

[98] Tutt ANJ, Garber JE, Kaufman B, Viale G, Fumagalli D, Rastogi P et al (2021) Adjuvant olaparib for patients with BRCA1- or BRCA2-mutated breast cancer. N Engl J Med 384(25):2394–2405 34081848 10.1056/NEJMoa2105215 PMC9126186

[99] Chan A, Moy B, Mansi J, Ejlertsen B, Holmes FA, Chia S et al (2021) Final efficacy results of neratinib in her2-positive hormone receptor-positive early-stage breast cancer from the phase III extenet trial. Clin Breast Cancer 21(1):80-91.e7 33183970 10.1016/j.clbc.2020.09.014

[100] Alemán JO, Farooki A, Girotra M (2014) Effects of tyrosine kinase inhibition on bone metabolism: untargeted consequences of targeted therapies. Endocr.-Related Cancer 21(3):R247–R259 10.1530/ERC-12-0400 24478055

[101] O’Sullivan S, Lin JM, Watson M, Callon K, Tong PC, Naot D et al (2011) The skeletal effects of the tyrosine kinase inhibitor nilotinib. Bone 49(2):281–289 21550432 10.1016/j.bone.2011.04.014

[102] O’Sullivan S, Horne A, Wattie D, Porteous F, Callon K, Gamble G et al (2009) Decreased bone turnover despite persistent secondary hyperparathyroidism during prolonged treatment with imatinib. J Clin Endocrinol Metab 94(4):1131–1136 19174494 10.1210/jc.2008-2324

[103] Fleissig Y, Regev E, Lehman H (2012) Sunitinib related osteonecrosis of jaw: a case report. Oral Surg, Oral Med, Oral Pathol and Oral Radiol 113(3):e1-3 10.1016/j.tripleo.2011.06.023 22676833

[104] Garuti F, Camelli V, Spinardi L, Bucci L, Trevisani F (2016) Osteonecrosis of THE JAW DURING SORAFENIB THERAPY FOR HEPATOCELLULAR CARCINOma. Tumori 102:S69-70 10.5301/tj.5000504 27079903

[105] Terpos E, Apperley J, Milojkovic D (2013) Imatinib and chronic myeloid leukemia: close to the bone. Leuk Lymphoma 54(8):1581–1582 23383598 10.3109/10428194.2013.772608

[106] Canzano F, Di Lella F, Manuguerra R, Vincenti V (2019) Osteonecrosis of the external auditory canal associated with oral sorafenib therapy: sorafenib and temporal bone osteonecrosis. Otol & Neurotol 40(8):e812 10.1097/MAO.0000000000002344 31356482

[107] Hosokawa T, Hara T, Arakawa Y, Oguma E, Yamada Y (2020) Periosteal reaction possibly induced by pazopanib: a case report and literature review. J Pediatr Hematol Oncol 42(8):e822 31567787 10.1097/MPH.0000000000001595

[108] Ramzan M, Verma R, Chopra YR, Yadav SP (2013) Post-fracture excessive callus formation in a child on imatinib therapy. Pediatr Blood Cancer 60(12):2087–2088 24038762 10.1002/pbc.24674

[109] Maita S, Yuasa T, Tsuchiya N, Mitobe Y, Narita S, Horikawa Y et al (2012) Antitumor effect of sunitinib against skeletal metastatic renal cell carcinoma through inhibition of osteoclast function. Int J Cancer 130(3):677–684 21387300 10.1002/ijc.26034

[110] Pokhrel NK, Kim YG, Kim HJ, Kim HJ, Lee JH, Choi SY et al (2019) A novel Bruton’s tyrosine kinase inhibitor, acalabrutinib, suppresses osteoclast differentiation and Porphyromonas gingivalis lipopolysaccharide-induced alveolar bone resorption. J Periodontol 90(5):546–554 30387495 10.1002/JPER.18-0334

[111] Ihn HJ, Kim JA, Bae YC, Shin HI, Baek MC, Park EK (2017) Afatinib ameliorates osteoclast differentiation and function through downregulation of RANK signaling pathways. BMB Rep 50(3):150–155 28256196 10.5483/BMBRep.2017.50.3.223 PMC5422028

[112] Campone M, Bondarenko I, Brincat S, Hotko Y, Munster PN, Chmielowska E et al (2012) Phase II study of single-agent bosutinib, a Src/Abl tyrosine kinase inhibitor, in patients with locally advanced or metastatic breast cancer pretreated with chemotherapy. Ann Oncol 23(3):610–617 21700731 10.1093/annonc/mdr261

[113] Giona F, Mariani S, Gnessi L, Moleti ML, Rea M, De Vellis A et al (2013) Bone metabolism, growth rate and pubertal development in children with chronic myeloid leukemia treated with imatinib during puberty. Haematologica 98(3):e25–e27 22983586 10.3324/haematol.2012.067447 PMC3659938

[114] Hoehn D, Cortes JE, Medeiros LJ, Jabbour EJ, Hidalgo JE, Kanagal-Shamanna R et al (2016) Multiparameter analysis of the “Off-Target Effects” of dasatinib on bone homeostasis in patients with newly diagnosed chronic myelogenous leukemia. Clin Lymphoma Myeloma Leuk 16(Suppl):S86-92 27521332 10.1016/j.clml.2016.02.027 PMC5843379

[115] Nguyen HM, Ruppender N, Zhang X, Brown LG, Gross TS, Morrissey C et al (2013) Cabozantinib Inhibits Growth of Androgen-Sensitive and Castration-Resistant Prostate Cancer and Affects Bone Remodeling. PLoS ONE 8(10):e78881 24205338 10.1371/journal.pone.0078881 PMC3808282

[116] Moseley KF, Naidoo J, Bingham CO, Carducci MA, Forde PM, Gibney GT et al (2018) Immune-related adverse events with immune checkpoint inhibitors affecting the skeleton: a seminal case series. J Immunother Cancer 6(1):104 30305172 10.1186/s40425-018-0417-8 PMC6180387

[117] Ye C, Lee K, Leslie WD, Lin M, Walker J, Kolinsky M (2023) Fracture rate increases after immune checkpoint inhibitor treatment: a potential new immune related adverse event. Osteoporos Int 34(4):735–740 36729143 10.1007/s00198-023-06690-1

[118] Johnson, Rachel. (2023) PD-1 blockade disrupts bone microarchitecture and compromises bone strength in breast cancer bone metastasis. Vancouver, ASBMR meeting.

[119] Gnant M, Baselga J, Rugo HS, Noguchi S, Burris HA, Piccart M et al (2013) Effect of everolimus on bone marker levels and progressive disease in bone in BOLERO-2. J Natl Cancer Inst 105(9):654–663 23425564 10.1093/jnci/djt026 PMC6430494

[120] Browne AJ, Kubasch ML, Göbel A, Hadji P, Chen D, Rauner M et al (2017) Concurrent antitumor and bone-protective effects of everolimus in osteotropic breast cancer. Breast Cancer Res 19:92 28793923 10.1186/s13058-017-0885-7 PMC5551016

[121] Kakiuchi Y, Yurube T, Kakutani K, Takada T, Ito M, Takeoka Y et al (2019) Pharmacological inhibition of mTORC1 but not mTORC2 protects against human disc cellular apoptosis, senescence, and extracellular matrix catabolism through Akt and autophagy induction. Osteoarthr Cartil 27(6):965–976 10.1016/j.joca.2019.01.009 30716534

[122] McDevitt RL, Quinlan C, Hersberger K, Sahai V (2018) Bone marrow edema associated with everolimus. Am JHealth-Sys Pharm 75(1):e23–e27 10.2146/ajhp170269 29273609

[123] Holzer G, Obermair A, Koschat M, Preyer O, Kotz R, Trieb K (2001) Concentration of vascular endothelial growth factor (VEGF) in the serum of patients with malignant bone tumors. Med Pediatr Oncol 36(6):601–604 11344490 10.1002/mpo.1136

[124] Zhang Q, Dong G, Wang F, Ding W (2020) Correlation between the changes of serum COX 2, APE1, VEGF, TGF-β and TSGF levels and prognosis in patients with osteosarcoma before and after treatment. J Cancer Res Ther 16(2):335–342 32474521 10.4103/jcrt.JCRT_11_20

[125] Bachelier R, Confavreux C, Peyruchaud O, Croset M, Goehrig D, Pluijm G (2014) Combination of anti-angiogenic therapies reduces osteolysis and tumor burden in experimental breast cancer bone metastasis. Int J Cancer 6(135):1319–1329 10.1002/ijc.28787 24615579

[126] Bettini G, Blandamura S, Saia G, Bedogni A. Bevacizumab-related osteonecrosis of the mandible is a self-limiting disease process. BMJ Case Rep. 2012; 10.1136/bcr-2012-007284 PMC4543697 23093510

[127] Pakosch D, Papadimas D, Munding J, Kawa D, Kriwalsky M (2013) Osteonecrosis of the mandible due to anti-angiogenic agent, bevacizumab. Oral Maxillofac Surg 4(17):303–306 10.1007/s10006-012-0379-9 23242941

[128] Nakagawa M, Kaneda T, Arakawa T, Morita S, Sato T, Yomada T et al (2000) Vascular endothelial growth factor (VEGF) directly enhances osteoclastic bone resorption and survival of mature osteoclasts. FEBS Lett 473(2):161–164 10812066 10.1016/s0014-5793(00)01520-9

[129] Bolomsky A, Schreder M, Meißner T, Hose D, Ludwig H, Pfeifer S et al (2014) Immunomodulatory drugs thalidomide and lenalidomide affect osteoblast differentiation of human bone marrow stromal cells in vitro. Exp Hematol 42(7):516–525 24704163 10.1016/j.exphem.2014.03.005

[130] Anderson G, Gries M, Kurihara N, Honjo T, Anderson J, Donnenberg V et al (2006) Thalidomide derivative CC-4047 inhibits osteoclast formation by down-regulation of PU.1. Blood 107(8):3098–3105 16373662 10.1182/blood-2005-08-3450

[131] Bolzoni M, Storti P, Bonomini S, Todoerti K, Guasco D, Toscani D et al (2013) Immunomodulatory drugs lenalidomide and pomalidomide inhibit multiple myeloma-induced osteoclast formation and the RANKL/OPG ratio in the myeloma microenvironment targeting the expression of adhesion molecules. Exp Hematol 41(4):387-397.e1 23178378 10.1016/j.exphem.2012.11.005

[132] Breitkreutz I, Raab MS, Vallet S, Hideshima T, Raje N, Mitsiades C et al (2008) Lenalidomide inhibits osteoclastogenesis, survival factors and bone-remodeling markers in multiple myeloma. Leukemia 22(10):1925–1932 18596740 10.1038/leu.2008.174

[133] Casali PG, Bielack S, Abecassis N, Aro HT, Bauer S, Biagini R et al (2018) Bone sarcomas: ESMO-PaedCan-EURACAN Clinical Practice Guidelines for diagnosis, treatment and follow-up. Ann Oncol 29:iv79-95 30285218 10.1093/annonc/mdy310

[134] Gaspar N, Hawkins DS, Dirksen U, Lewis IJ, Ferrari S, Le Deley MC et al (2015) Ewing sarcoma: current management and future approaches through collaboration. J Clin Oncol 33(27):3036–3046 26304893 10.1200/JCO.2014.59.5256

[135] Hayward R, Iwaniec UT, Turner RT, Lien CY, Jensen BT, Hydock DS et al (2013) Voluntary wheel running in growing rats does not protect against doxorubicin-induced osteopenia. J Pediatr Hematol Oncol 35(4):e144-148 23211689 10.1097/MPH.0b013e318279b1fb

[136] Holzer G, Hobusch G, Hansen S, Fischer L, Patsch JM (2021) Is There an Association Between Bone Microarchitecture and Fracture in Patients who were Treated for High-grade Osteosarcoma? A controlled study at long-term follow-up using high-resolution peripheral quantitative CT. Clin Orthop Relat Res 479(11):2493–2501 34077400 10.1097/CORR.0000000000001842 PMC8509943

[137] Pirker-Frühauf UM, Friesenbichler J, Urban EC, Obermayer-Pietsch B, Leithner A (2012) Osteoporosis in children and young adults: a late effect after chemotherapy for bone sarcoma. Clin Orthop Relat Res 470(10):2874–2885 22806259 10.1007/s11999-012-2448-7 PMC3441998

[138] Song D, Meng T, Xu W, Hou T, Lin Z, Yin H et al (2015) 5-Fluoruracil blocked giant cell tumor progression by suppressing osteoclastogenesis through NF-kappaB signals and blocking angiogenesis. Bone 78:46–54 25956534 10.1016/j.bone.2015.04.047

