# Supplementary material for: Bone Effects of Anti-Cancer Treatments in 2024
Source: Calcif Tissue Int. 2025 Mar 27;116(1):54. doi: 10.1007/s00223-025-01362-0 (PMC11950069; doi:10.1007/s00223-025-01362-0)
Supplement: Supplementary file 1 — Supplementary file1 (DOCX 2897 KB) [file 223_2025_1362_MOESM1_ESM.docx]

**Supplemental table 1** Molecular targets of tyrosine kinase inhibitors in clinical cancer field.

| TKI inhibitor | cdk4,6 | btk | erbb | alk | ret | pik alpha | vegfr | egfr | bcr-abl | scr | c-kit | pdgf | ros | igf-1r | met | raf | trk | vgfr | flt3 | braf |
| --- | --- | --- | --- | --- | --- | --- | --- | --- | --- | --- | --- | --- | --- | --- | --- | --- | --- | --- | --- | --- |
| Abemaciclib | x |  |  |  |  |  |  |  |  |  |  |  |  |  |  |  |  |  |  |  |
| Acalabrutinib |  | x |  |  |  |  |  |  |  |  |  |  |  |  |  |  |  |  |  |  |
| Afatinib |  |  | x |  |  |  |  |  |  |  |  |  |  |  |  |  |  |  |  |  |
| Alectinib |  |  |  | x | x |  |  |  |  |  |  |  |  |  |  |  |  |  |  |  |
| Alpelisib |  |  |  |  |  | x |  |  |  |  |  |  |  |  |  |  |  |  |  |  |
| Axitinib |  |  |  |  |  |  | x |  |  |  |  |  |  |  |  |  |  |  |  |  |
| Bosutinib |  |  |  |  |  |  |  |  | x | x | x | x |  |  |  |  |  |  |  |  |
| Brigatinib |  |  |  | x |  |  |  |  |  |  |  |  | x | x |  |  |  |  |  |  |
| Cabozantinib |  |  |  |  |  |  | x |  |  |  |  |  |  |  | x |  |  |  |  |  |
| Ceritinib |  |  |  | x |  |  |  |  |  |  |  |  |  |  |  |  |  |  |  |  |
| Cobimetinib |  |  |  |  |  |  |  |  |  |  |  |  |  |  | x |  |  |  |  |  |
| Crizotinib |  |  |  | x |  |  |  |  |  |  |  |  |  |  |  |  |  |  |  |  |
| Dabrafenib |  |  |  |  |  |  |  |  |  |  |  |  |  |  |  | x |  |  |  |  |
| Dasatinib |  |  |  |  |  |  |  |  | x | x | x |  |  |  |  |  |  |  |  |  |
| Entrectinib |  |  |  | x |  |  |  |  |  |  |  |  | x |  |  |  | x |  |  |  |
| Erdafitinib |  |  |  |  |  |  |  |  |  |  |  |  |  |  |  |  |  | x |  |  |
| Erlotinib |  |  |  |  |  |  |  | x |  |  |  |  |  |  |  |  |  |  |  |  |
| Gefitinib |  |  |  |  |  |  |  | x |  |  |  |  |  |  |  |  |  |  |  |  |
| Ibrutinib |  | x |  |  |  |  |  |  |  |  |  |  |  |  |  |  |  |  |  |  |
| Imatinib |  |  |  |  |  |  |  |  | x |  | x |  |  |  |  |  |  |  |  |  |
| Lapatinib |  |  |  |  |  |  |  | x |  |  |  |  |  |  |  |  |  |  |  |  |
| Larotrectinib |  |  |  |  |  |  |  |  |  |  |  |  |  |  |  |  | x |  |  |  |
| Lenvatinib |  |  |  |  | x |  | x |  |  |  | x | x |  |  |  |  |  | x |  |  |
| Lorlatinib |  |  |  | x |  |  |  |  |  |  |  |  | x |  |  |  |  |  |  |  |
| Midostaurine |  |  |  |  |  |  |  |  |  |  | x |  |  |  |  |  |  |  | x |  |
| Nilotinib |  |  |  |  |  |  |  |  |  | x | x | x |  |  |  |  |  |  |  |  |
| Osimertinib |  |  |  |  |  |  |  | x |  |  |  |  |  |  |  |  |  |  |  |  |
| Palbociclib | x |  |  |  |  |  |  |  |  |  |  |  |  |  |  |  |  |  |  |  |
| Pazopanib |  |  |  |  |  |  | x |  |  |  | x | x |  |  |  |  |  |  |  |  |
| Ponatinib |  |  |  |  |  |  |  |  | x |  |  |  |  |  |  |  |  |  |  |  |
| Pralsetinib |  |  |  |  | x |  |  |  |  |  |  |  |  |  |  |  |  |  |  |  |
| Regorafenib |  |  |  |  |  |  | x |  |  |  |  |  |  |  |  | x |  |  |  |  |
| Ribociclib | x |  |  |  |  |  |  |  |  |  |  |  |  |  |  |  |  |  |  |  |
| Ripretinib |  |  |  |  |  |  | x |  |  |  | x | x |  |  |  |  |  |  |  | x |
| Saracatinib |  |  |  |  |  |  |  |  | x | x |  |  |  |  |  |  |  |  |  |  |
| Sorafenib |  |  |  |  |  |  | x |  |  |  | x |  |  |  |  | x |  |  |  |  |
| Sunitinib |  |  |  |  |  |  | x |  |  |  | x | x |  |  |  |  |  |  |  |  |
| Trametinib |  |  |  |  |  |  |  |  |  |  |  |  |  |  | x |  |  |  |  |  |
| Vandetanib |  |  |  |  | x |  | x | x |  |  |  |  |  |  |  |  |  |  |  |  |
| Vemurafenib |  |  |  |  |  |  |  |  |  |  |  |  |  |  |  |  |  |  |  | x |
